# Genomic analysis of atypical fibroxanthoma

**DOI:** 10.1371/journal.pone.0188272

**Published:** 2017-11-15

**Authors:** Kevin Lai, Catherine A. Harwood, Karin J. Purdie, Charlotte M. Proby, Irene M. Leigh, Namita Ravi, Thaddeus W. Mully, Lionel Brooks, Priscilla M. Sandoval, Michael D. Rosenblum, Sarah T. Arron

**Affiliations:** 1 Department of Dermatology, University of California, San Francisco, California, United States of America; 2 Center for Cutaneous Research and Cell Biology, Barts and the London School of Medicine and Dentistry, Queen Mary University of London, London, United Kingdom; 3 Division of Cancer Research, School of Medicine, University of Dundee, Dundee, United Kingdom; 4 Veterans Administration Medical Center, San Francisco, California, United States of America; German Cancer Research Center (DKFZ), GERMANY

## Abstract

Atypical fibroxanthoma (AFX), is a rare type of skin cancer affecting older individuals with sun damaged skin. Since there is limited genomic information about AFX, our study seeks to improve the understanding of AFX through whole-exome and RNA sequencing of 8 matched tumor-normal samples. AFX is a highly mutated malignancy with recurrent mutations in a number of genes, including *COL11A1*, *ERBB4*, *CSMD3*, and *FAT1*. The majority of mutations identified were UV signature (C>T in dipyrimidines). We observed deletion of chromosomal segments on chr9p and chr13q, including tumor suppressor genes such as *KANK1* and *CDKN2A*, but no gene fusions were found. Gene expression profiling revealed several biological pathways that are upregulated in AFX, including tumor associated macrophage response, GPCR signaling, and epithelial to mesenchymal transition (EMT). To further investigate the presence of EMT in AFX, we conducted a gene expression meta-analysis that incorporated RNA-seq data from dermal fibroblasts and keratinocytes. Ours is the first study to employ high throughput sequencing for molecular profiling of AFX. These data provide valuable insights to inform models of carcinogenesis and additional research towards tumor-directed therapy.

## Introduction

Atypical fibroxanthoma (AFX) is a rare cutaneous neoplasm that typically affects older white males with a history of sun exposure or radiation. Although precise epidemiologic data are lacking, AFX comprises less than 1% of skin cancers removed by Mohs micrographic surgery[1]. Histologically, AFX demonstrates marked variety in cellular composition, including bizarre, pleomorphic macrophages, spindled fibroblasts, and epithelioid cells. The histologic differential diagnosis includes poorly-differentiated squamous cell carcinoma (cSCC), desmoplastic melanoma, and soft tissue sarcomas such as leiomyosarcoma and undifferentiated pleomorphic sarcoma/ malignant fibrous histiocytoma (UPS/MFH). AFX is a diagnosis of exclusion based on negative immunohistochemistry to rule out other cancers[2]. AFX may stain with CD68, vimentin, actin, or CD10, though these are not consistent nor specific to the diagnosis[3].

Many sarcomas are driven by gene fusions, such as *COL11A1-PDGF* that drives dermatofibrosarcoma protuberans (DFSP)[4]. However, fusions have not been investigated in AFX. It has also been suggested that the spindled cells in AFX are not dermal-derived fibroblasts but rather malignant keratinocytes that have undergone epithelial-to-mesenchymal transition (EMT)[5,6].

Targeted studies have identified genetic aberrations in AFX, including mutations in the coding region of *TP53* [7] and *TERT* promoter[8], and deletions of chr9p and chr13q[9]. To characterize the genomic landscape of AFX, we sequenced the exomes and transcriptomes of 8 tumors and paired normal control skin biopsies. We investigated gene fusions with Nugen’s Ovation Fusion Panel Target Enrichment System. Our analyses identified recurrent mutated genes and copy number variations (CNV) as well as gene pathways involved in tumor-associated macrophage (TAM) response and EMT.

## Methods

### Sample collection

Eight tumors were selected for inclusion. Eligible tumors were superficial cutaneous papules or nodules, diagnosed by skin biopsy with formalin fixation and paraffin embedding. The diagnosis was based on routine histopathology including dermal proliferation of spindled cells and/or pleomorphic multinucleate giant cells, with numerous atypical cells and mitoses. Eligible tumors did not express S100 or Sox-10, did not express keratin, but did label with CD10 and procollagen-1. Fresh tissue was obtained for this study from debulking specimens prior to Mohs micrographic surgery at UCSF and the San Francisco VA Medical Center (7 specimens) or wide local excision at Barts Health NHS Trust (1 specimen), and histopathologic features were confirmed on frozen section. There were no findings of deeper invasion, or perineural/perivascular invasion.

Tumors and peritumoral normal skin from both centers were snap frozen, embedded in Tissue-Tek (Sakura Finetek, US) and stored at -80°C. All eight specimens were processed and sequenced together to avoid batch effect inconsistencies. To enrich for cell populations, 8um sections were cut onto 1.0mm PEN membrane slides (Zeiss, Cambridge, UK) and laser-capture microdissection of tumor and normal tissue was performed using the Zeiss Palm Microbeam microscope (Zeiss). QIAamp DNA micro and RNeasy micro kits were used for nucleic acid extraction (Qiagen, Crawley, UK). All subjects that contributed tumor for exome/transcriptome sequencing signed written informed consent under protocols approved by the UCSF Institutional Review Board or the East London and City Health Authority Local Ethics Committee, in accordance with the Declaration of Helsinki Principles. De-identified tumors for flow cytometry were collected as anonymous surgical discard specimens and self-certified by the investigators as “Not Human Subjects Research” per UCSF IRB guidelines.

### Exome sequencing

Exome sequence libraries were batch prepared with the Nimblegen SeqCap EZ Exome 3.0 capture kit and libraries sequenced in one run on the Illumina HiSeq 2500 for 101-basepair, paired-end reads. Sequencing results were quality checked using FastQC v0.11.1. Sequences were aligned to the human genome (hg19) using bwa-mem v0.7.12[10]. GATK pipeline was used to prep the BAM files for variant calling. Picard v1.129 marked PCR duplicates, GATK v3.4.0[11] realigned the reads around Indels and recalibrated the base scores, and Samtools v1.2[12] sorted and indexed the aligned bam files.

Mutect v1.1.7[13], Varscan2 v2.3.8[14], and Strelka v1.0.14[15] were used to find somatic single-nucleotide variants (SNVs) and Indels. Only mutations detected in two of three programs were kept. Results were annotated using Oncotator v1.5.3.0[16]. A python script was written to determine the number of C>T mutations that appeared in dipyrimidines. For driver mutation discovery, OncodriveFM v0.6.0[17], OncodriveClust v0.4.1[18], and MutsigCV v1.4[19] were used with default parameters. CNVKit v0.6.1[20] was run to identify CNV using the processed bam files and SeqCap EZ 3.0 exome capture file. These data have been deposited to the SRA (Short Read Archive) database under SRP082197.

### Gene fusion detection

Nugen Ovation Fusion Panel Target Enrichment System was used to identify potential gene fusions. Two DFSP positive controls were prepared in parallel. A pre-defined pool of 500 genes from COSMIC and ChimerDB are included in the fusion panel. After sequencing on an Illumina 2500, Chimerascan v0.4.5[21] and Soapfuse v1.26[22] were used to identify gene fusions from the sequencing results using default parameters. Nested PCR primers were designed to span potential fusions for verification.

### Transcriptome sequencing

RNA samples were reverse transcribed with Nugen Ovation RNA-Seq system kit. Libraries were batch prepared from fragmented cDNA using Nugen Ovation Ultralow kit, and sequenced on the Illumina HiSeq 2500 in one run. Resulting reads were aligned to the human genome (hg19) using Tophat v2.0.14[23], and htseq-count v0.6.0[24] obtained counts using the refflat file downloaded from the UCSC genome browser. To obtain differential expression, edgeR v3.10.5[25] was run accounting for the tumor-normal patient pairs. Genes with at least 1 count per million (CPM) in 1 sample were considered for differential expression. Four publicly-available dermal fibroblast datasets from the SRA were downloaded (SRR1976435, SRR1976434, SRR1976433, and SRR773861), and processed in parallel. EdgeR determined DEG between the AFX tumors and these samples. The CPM table was input into Cluster v3.0[26] to perform hierarchal clustering using mean-center, normalizing genes, and average linkage. GSEA (Gene Set Enrichment Analysis)[27], was used to perform Gene Ontology analysis. Cluster files were viewed on Gene Pattern’s Hierarchical Clustering Viewer[28]. The R package prcomp v3.2.2 performed the PCA with log CPM values, and ggbiplot v0.55 graphed the PCA results. For t-SNE analysis, scaled estimates for each cancer was downloaded from the firebrowse repository. Scaled estimates were converted to TPM (transcripts per million). RSEM was run on AFX samples to generate TPM counts. The R package Rtsne v0.11 performed the t-SNE analysis with these counts, and plotted using ggplot2 v2.1.0. RNASeq data has been deposited to the GEO (Gene Expression Omnibus) database under GSE85671.

### Flow cytometry

Multi-parameter flow cytometry was performed on samples obtained from tumor and adjacent non-tumor tissue as previously described [29]. Freshly isolated samples were minced and digested overnight with buffer consisting of Collagenase Type 4 (Worthington LS004188), DNAse (Sigma DN25-1G), 10% FBS, 1% HEPES, and 1% Penicillin/Streptavidin in RPMI 1640 medium. Single cell suspensions were filtered, centrifuged, and counted. Approximately 2x10^6^ cells were stained with multiple fluorochrome-conjugated monoclonal antibodies. The following antibodies were used: anti-human CD45 (anti-hCD45) (H130; eBioscience), anti-hCD16 (CB16; eBioscience), anti-hCD14 (M5E2; BD Biosciences), anti-hCD169 (7–239; Biolegend), anti-hCD206 (19.2; BD Biosciences), anti-hCD1c (AD5-8E7; MACS), anti-hCD163 (GHI/61; BD Biosciences), anti-hCD68 (Y1/82A; BD Biosciences), anti-HLA-DR (2243; eBioscience), and Ghost Dye Violet 510 (Tonbo biosciences). Data was acquired by an LSRFortessa (BD Biosciences) and analyzed using FlowJo software (Tree Star, Inc.).

## Results

### Histopathology

**Fig 1:**
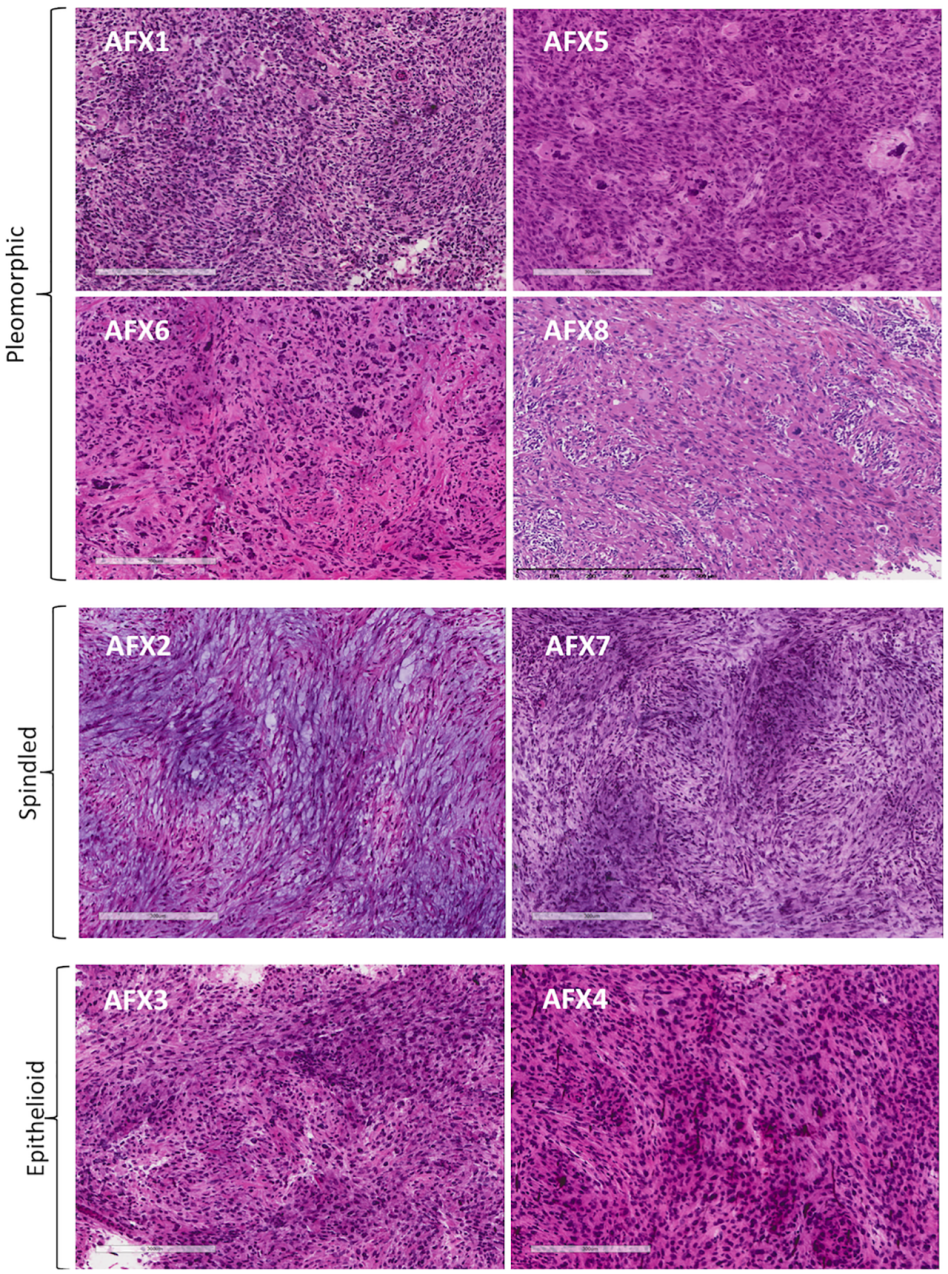
Histopathology of AFX. AFX samples were microdissected from snap-frozen, OCT-embedded surgical specimens. The spectrum of predominant cytomorphology included four pleomorphic/histocytic (AFX 1, 5, 6, and 8), two spindled (AFX 2 and 7), and two epithelioid (AFX 3 and 4). Scale bar indicates 300μm.

Eight tumors were identified for sequencing (Fig 1). Four tumors demonstrated marked pleomorphism, with bizarre macrophages, nuclear atypia, and multinucleate giant cells. Two had predominantly spindled histology, with whorls and fascicles of densely packed fibroblasts with spindled nuclei. Two had epithelioid histology, with rounder cells and plump nuclei. We observed varying levels of lymphocytic infiltrate, predominantly in the spindled and epithelioid subtypes.

### Exome sequencing

**Fig 2:**
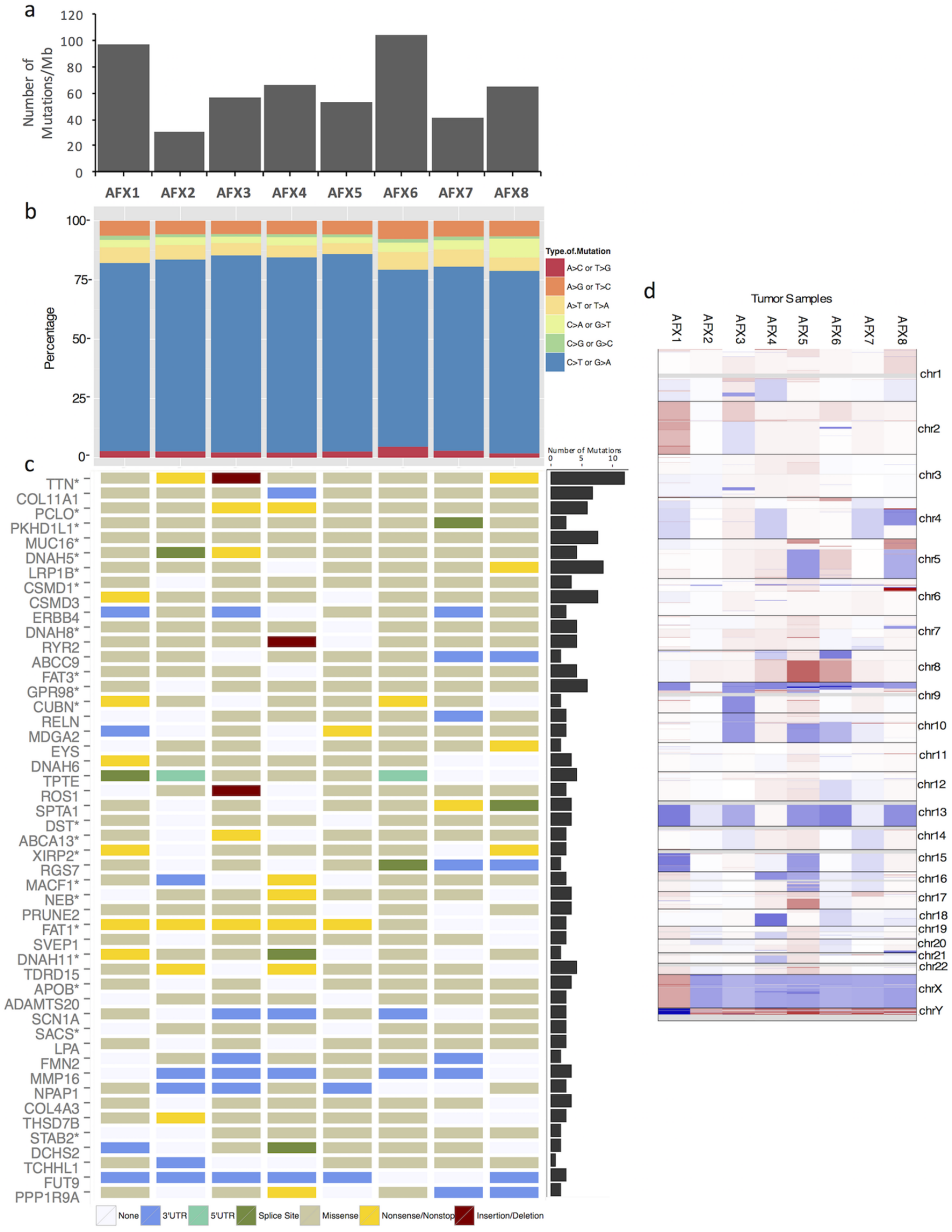
Whole exome sequencing identifies copy number variations, and frequent UV mutations in AFX. **(a)** Histogram of somatic mutation rate (number of mutations/megabase of DNA) for each AFX tumor. **(b)** Stacked plot of the percentage of mutations of each type. **(c)** Matrix illustrates genes that are mutated in at least 75% of the AFX tumors and the type of mutations found. When more than one mutation is present for a single gene, only one type of mutation is shown delineated in order by the legend; insertion/deletion, nonsense/nonstop, missense, splice-site, 3’ or 5’UTR. **(d)** A heatmap of copy number variations in the 8 tumor samples relative to the normal. Red represents gains, blue represents deletions, and white represents no losses or gains in that location in the genome.

We performed exome sequencing on eight AFX tumors and patient-paired normal keratinocytes. An average of 138 million reads/sample were obtained, with average alignment rate of 99.31%. Mean coverage across targeted bases was 113X for AFX and 62X for normal samples, with 58% of targeted bases having at least 20X coverage. We employed multiple bioinformatics tools to optimize specificity for mutation calling[30]. Mutect, Varscan2, and Strelka detected an average of 2945, 4930, and 4740 **SNVs** respectively. Varscan2 and Strelka detected an average of 466 and 30 insertions/deletions (Indels). **SNVs** found in two of three programs (4128 on average) and Indels found in both programs (22 on average) were annotated using Oncotator for downstream analyses (S1 Table). AFX has a very high mutational burden with a somatic mutation rate of 64 mutations per megabase of DNA (Fig 2a). The COSMIC database was used to query mutations in canonical cancer genes, however no recurrent SNP or Indels was identified in our tumors.

Ultraviolet (UV)-induced mutations are characterized by C>T transitions in dipyrimidines[31] and play a key role in skin cancer development[32]. More than 70% of mutations in AFX were C->T mutations in dipyrimidines (Fig 2b), suggesting that AFX are driven by UV radiation.

A previous comparative genomic hybridization (CGH) array study reported deletions in chr9p and chr13q[9]. We evaluated CNV in our samples using CNVKit. In the majority of samples, we confirmed deletions in chr9 and chr13 (Fig 2d). Chr9 had larger magnitude copy number deletions than chr13, and there were 80 deleted genes in chr9 in 87% of tumors (S2 Table). Tumor suppressors *KANK1* (-0.5332 average log2 copy ratio (CR)) and *CDKN2A* (-1.0184 average log2 CR) were deleted in chr9. *MTAP*, a gene frequently co-deleted with *CDKN2A*, was deleted in our samples (-1.0184 average log2 CR).

### Commonly mutated genes in AFX

The mutational signature of AFX is shown in Fig 2c. Of 49 genes mutated in more than 75% of samples, 21 were identified as FLAGS, a term describing genes that appear frequently in a majority of exome sequencing studies and may not have clinical significance[33]. Although FLAG genes are generally not considered potential drivers, 5/8 of our tumors had nonsense mutations in *FAT1* (average variant allele frequency (VAF) = 30.38%), leading to premature stop codons (Fig 2c). 4 of these had nonsense mutations located within the cadherin domain, and the fifth had a nonsense in the laminin G region. The most mutated non-FLAG gene was *COL11A1* (average VAF = 30.17%), with missense mutations in 7/8 AFX tumors and a 3’UTR mutation in the eighth. Other top mutated non-FLAG genes include *CSMD3* (average VAF = 30.24%) and *ERBB4* (average VAF = 25.21%).

### RNA fusion sequencing

To identify potential gene fusions, we used Nugen Ovation Fusion Panel Target Enrichment System for RNA library preparation prior to high-throughput sequencing[34]. We used Chimerascan and Soapfuse to identify gene fusions from the sequencing results. *COL1A1-PDGFB* fusions were readily detected in two control DFSP, with spanning reads present at 600–1000 times any of the potential fusions detected in AFX. We were unable to validate any of the top AFX fusion candidates from these programs with nested PCR, suggesting false positive computational results (data not shown).

### Transcriptome sequencing

**Fig 3:**
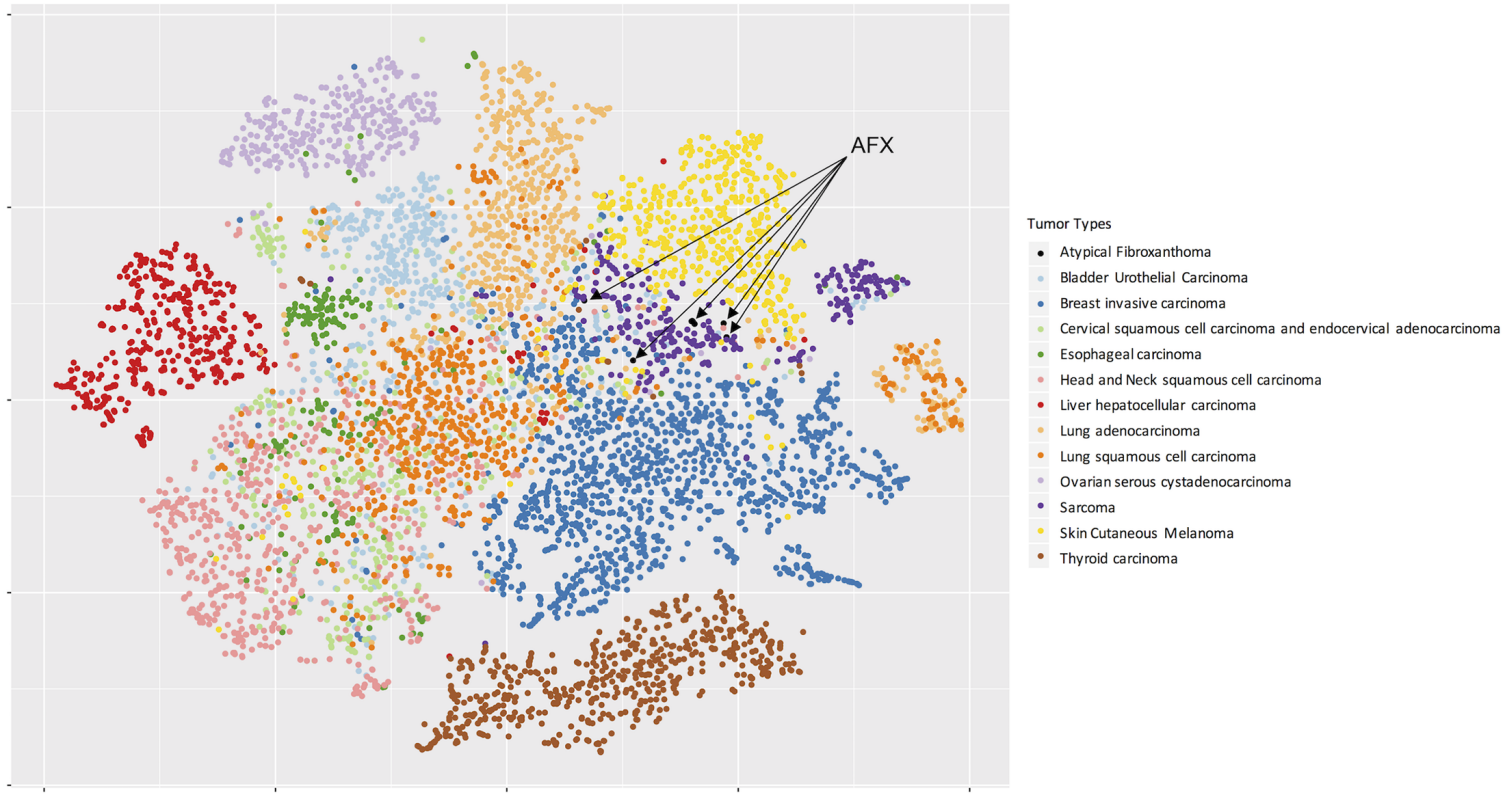
AFX gene expression clusters with other sarcomas. T-SNE plot of 8 AFX tumors with publicly available tumors from TCGA. AFX are highlighted as black dots, clustering within other sarcomas (colored purple).

We performed RNA-seq on the same set of matched tumor-normal pairs. We obtained an average of 67.5 million 101bp paired-end reads per sample. As normal dermal fibroblasts were not available from these patients, we used publicly-available fibroblast RNA-seq expression data[35] for comparison. Principal component analysis (PCA) between tumors, matched normal keratinocytes, and non-matched dermal fibroblasts was largely driven by differences between normal keratinocytes and fibroblasts, with tumors falling between the two cell types on PC1 (S1 Fig). PC2 reflected expression differences between normal cells of both types and tumor tissue. Global gene expression in AFX was compared to publicly available expression through The Cancer Genome Atlas (TCGA). AFX expression overlapped with other sarcomas, and otherwise fell closest to cutaneous melanoma (Fig 3).

We used edgeR paired analysis on the matched tumor-normal samples to identify differentially expressed genes (DEG) between AFX and keratinocytes. 8591 DEG were identified with a FDR < 0.05, of which 3524 genes had a log2 fold-change (FC) > |2|. To account for the possibility that these tumors are fibroblastic in origin, we ran edgeR (non-paired) against the publicly-available dermal fibroblast samples. This analysis identified 4884 DEG using a FDR cutoff of 0.05. We carried forward the intersection of both analyses (1446 DEG) for further investigation (S3 Table).

### Multiple signaling pathways are dysregulated in AFX

To investigate gene dysregulation in AFX, Gene Set Enrichment Analysis (GSEA) was used with the pre-ranked method on the 1446 DEG. There were 88 upregulated significantly enriched pathways, and 59 downregulated significantly enriched pathways among Kegg, Reactome, Biocarta, and Gene Ontology. Defense response, immune system, and GPCR ligand binding were the most enriched pathways (S2 Fig). GSEA also identified significantly upregulated *KRAS* signaling (MSigDB M5953; 0.001 FDR) and significantly downregulated *p53* signaling (MSigDB M5939; 0.164 FDR). Several *WNT* pathway genes were identified as significantly differentially expressed including *WISP1* (4.89 log2 fold change (FC)), *FZD1*(1.59 log2 FC), *PORCN* (1.46 log2 FC), and *SFRP*(-2.67 log2 FC).

### High tumor-associated macrophage response in AFX

**Fig 4:**
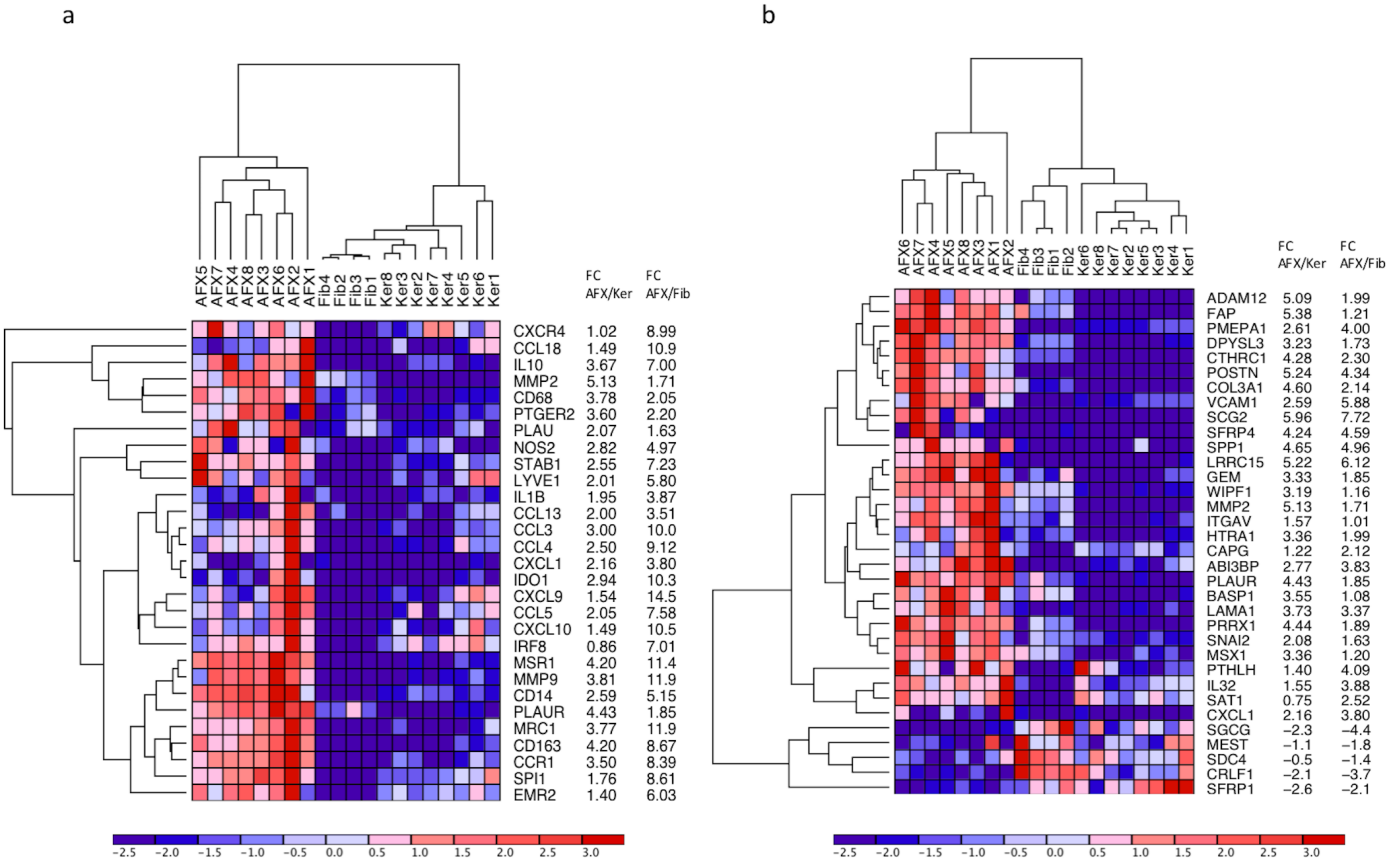
RNA sequencing reveals differentially expressed genes and pathways in AFX. (**a**) Heatmap of DE genes extracted from literature reviews that are associated with TAMs (higher expression is red, while lower expression is shown as blue). (**b**) Heatmap of genes from the Hallmark Epithelial Mesenychmal Transition Pathway from GSEA.

To investigate the immune infiltrate, we analyzed the top ranking genes in the immune system pathway (MSigDB M1045). These included *MRC1* (macrophage mannose receptor 1) and scavenger receptor (*MSR1*), providing evidence of an M2 macrophage population. The pattern of gene expression was characteristic of a TAM infiltrate (Fig 4a). Several genes involved with tumor macrophage recruitment[36] were significantly upregulated in AFX, including *CCL5*, *CCL3*, *CCL4*, *CCR1*, and *CCL18*. Other genes involved in tumor promotion and extracellular matrix remodeling were increased, including *MMP2*, *MMP9*, *IL-10*, and *IL1B*. (Fig 4a).

**Fig 5:**
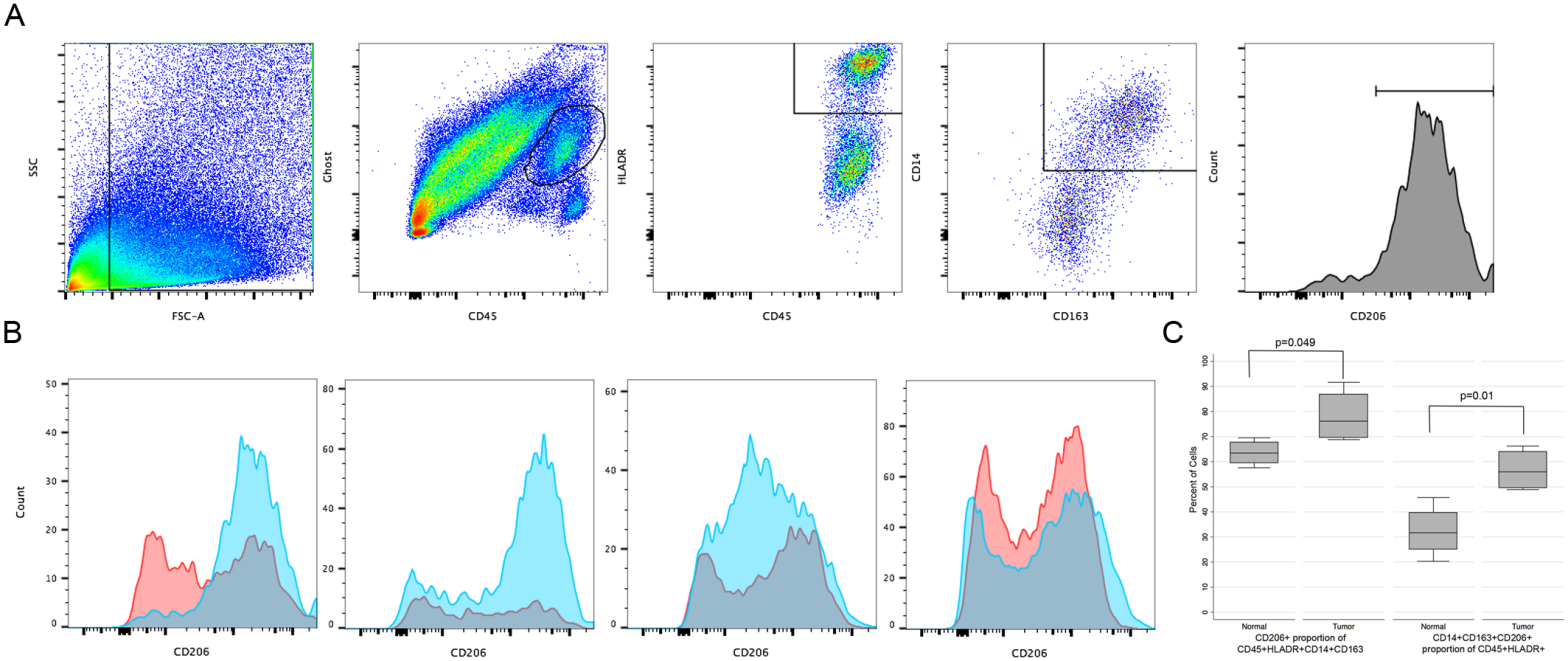
M2 tumor-associated macrophages (TAM) are enriched in AFX. A. Schematic of successive gating strategy of live myeloid cells expressing CD45 and HLADR. These were further gated on CD14+CD163+ to identify macrophages; CD206 expression was measured on this subset. A representative sample is shown. B. CD206 expression in four AFX (blue) and patient-matched normal skin (red). C. CD206+ TAM are enriched in AFX as a proportion of CD45+HLADR+CD14+CD163+ macrophages (left, p = 0.049); CD14+CD163+CD206+ macrophages are enriched as a proportion of CD45+HLADR+ myeloid cells (right, p = 0.01).

The TAM transcriptional signal was confirmed by flow cytometry on four additional primary AFX (Fig 5). CD206+ TAM were enriched in AFX as a proportion of CD45+HLADR+CD14+CD163+ macrophages; and CD14+CD163+CD206+ macrophages are enriched as a proportion of CD45+HLADR+ myeloid cells.

### EMT response in AFX

EMT is indicated by an upregulation of mesenchymal markers with a downregulation of epithelial markers[37]. PCA analysis indicated that overall gene expression of AFX tumors was located on a spectrum between keratinocytes and fibroblasts (S1 Fig). Hallmark of EMT (MSigDB M5930) pathway was highly upregulated with a normalized enrichment score of 2.89 and a FDR of 6.9E-5 (S2 Fig). *Slug*, a known inducer of EMT, was also upregulated in the AFX tumors. Slug contributed to the core enrichment of the EMT pathway with a log2 fold-change of 2.08 and a FDR of 1.22E-12. Other genes that contributed to the core enrichment are *POSTN* (5.24 log2 FC), *CTHRC1* (4.29 log2 FC), *WIPF1* (3.19 log2 FC), *ADAM12* (5.09 log2 FC), and *LAMA1*(3.73 log2 FC) (Fig 4b).

## Discussion

AFX is a rare neoplasm of uncertain cellular origin. Our aim was to describe genomic mutations and transcriptional changes in AFX, to lay the groundwork for larger studies in this rare tumor. Exome sequence analyses revealed a high UV mutational burden, similar to other skin cancers, including cSCC[38] and Merkel cell carcinoma[39]. A secondary aim of this work was to address the hypothesis that AFX is a fusion-driven dermal sarcoma. Given the prevalence of driver gene fusions in other sarcomas, we sought to identify fusion genes in AFX using a novel sequence-based method for gene fusion detection, but none were identified.

We identified frequently mutated genes in AFX, including *FAT1*, *COL11A1*, *CSMD3*, and *ERBB4*, and CNV analysis was consistent with prior report of deletions in chr9p and chr13q[9]. Somatic mutations in tumor suppressor *FAT1* have been linked to Wnt signaling, driving cancer development[40]. We identified nonsense *FAT1* mutations in 5/8 AFX tumors. Several *WNT* pathway genes were significantly differentially expressed in AFX including *WISP1*, *FZD1*, *PORCN*, and *SFRP*. These mutations and dysregulated genes suggest that *FAT1* and *WNT* signaling may play a role in the development or progression of AFX.

All eight AFX tumors had mutations in the structural collagen gene *COL11A1*, which is a poor prognostic marker in lung, head and neck, and ovarian cancer[41–43]. Mutation in *COL11A1* has been reported in up to 75% of cSCC[44], and is associated with higher genome mutation density in lung cancer [45], suggesting a role for genome destabilization. *CSMD3* was mutated in 87.5% of AFX. This gene is mutated in over 80% of cSCC[44], and was previously identified as the second most mutated gene in non-small cell lung cancer, regulating cell proliferation[46]. Mutations in both *CSMD3* and *COL11A1* are not specific to any one cancer type, and these genes may be accruing mutations due to their long length. Future mechanistic studies will be needed to see if *CSMD3* and *COL11A1* play a role in AFX.

*CDKN2A* and *MTAP* were co-deleted in AFX, and *MTAP* was significantly downregulated (-1.33 log2 FC). *CDKN2A* and *MTAP* are often co-deleted due to their close proximity in chr9p[47]. *p19ARF*, a product of *CDKN2A*, is known to block *MDM2*, resulting in dysregulation of the p53 pathway[48]. Inactivation of *CDKN2A* is also thought to play a role in the cSCC development[49]. Deletion of this locus, causing an inactivation of *CDKN2A*, could be the cause of the downregulation of the p53 pathway in our AFX. Downregulation of the p53 pathway, *CDKN1A* (-1.855 log2 FC) and *CDKN1B* (-0.899 FC) suggests aberrant cell cycle regulation.

RNA-seq analysis demonstrates expression-level similarities between AFX and other sarcomas, confirming the histopathologic classification. Despite finding no mutation or amplification of the *KRAS* gene, genes upregulated by *KRAS* activation were enriched in AFX. Average coverage of *KRAS* was 135X with 98% of targeted regions having at least 30X coverage, sufficient to detect mutation. No mutations in *KRAS* were identified in RNASeq data. Future studies will investigate the mechanism of *KRAS* upregulation, but this finding suggests that AFX may respond to therapy targeting Ras signaling.

Importantly, we observed upregulation of genes associated with TAM response in AFX compared to normal skin. TAMs closely resemble M2 macrophages, promoting tumor development and progression by activating pro-tumor immune responses, remodeling/degrading the extracellular matrix, and stimulating proliferation[50,36,51]. TAM response is common among cancers, but the bizarre macrophage pleomorphism in AFX suggests macrophage atypia driving hyperproliferation of dermal fibroblasts. Pleomorphic AFX may represent the early stage of carcinogenesis, followed by a longer-term spindled phase in which the macrophages are no longer required to maintain fibrosis. Future studies will be required to address this hypothesis, including investigation of individual cell types and the contribution of *CDKN2A*, *KRAS*, *AKT* and *FAT1* pathways in tumorigenesis. In addition, this tumor may serve as a model for understanding TAM response and profibrotic macrophage-driven inflammatory disease.

Our analyses also indicated an upregulation of EMT in AFX. EMT can be activated and promote tumor progression through a variety of different pathways, including the *AKT-PI3K*, *RAS*, *ERK*, *MAPK*, and *FGF* pathways[37]. It is curious to observe upregulation of EMT in a tumor that is presumably mesenchymal to begin with. However, the phenomenon is observed in other sarcomas such as Ewing sarcoma, in which a “metastable” phenotype arises between epithelial and mesenchymal states[52,53]. Transition between states is marked by shifts in EMT and mesenchymal to epithelial (MET) transition. Another potential explanation is that AFX is derived from a dedifferentiated keratinocyte carcinoma, and the EMT pathways observed result in fibrohistiocytic morphology. EMT is typically associated with progression and metastasis, which is rare in AFX compared to other sarcomas. However, AFX do have metastatic potential, with recurrence rates between 5–16% and metastasis rates from 1–6%[54]. Though this is lower than soft tissue sarcomas, it is higher than that of cutaneous squamous cell carcinoma and aligns with the intermediate histologic and transcriptional phenotype.

A limitation of this study is that normal keratinocytes were used as controls. Tissue available for microdissection did not yield adequate RNA for analysis of patient-paired normal fibroblasts. We addressed this by incorporating publicly-available fibroblast expression data. Another limitation is the small number of tumors available for sequencing. Exome data from a small number of rare tumors is typically not sufficient to identify driver mutations in cancer, with MutSigCV, OncodriveFM, or OncodriveClust. When using these programs with our dataset, *FAT1* was identified as a potential driver by MutSigCV and OncodriveFM (p-value < 0.05). However, the results were non-significant when accounting for multiple testing due to a lack of statistical power (data not shown). However, atypical fibroxanthoma is an extraordinarily rare tumor, and obtaining fresh tissue samples for sequencing is exceedingly difficult. Our hope is that the initial data obtained here can justify larger studies using focused analysis on archival tissue samples.

This work provides an initial genomic analysis of AFX and expands on previous studies in this rare tumor. Our data suggest that this tumor is not a fusion-driven sarcoma, but rather may be driven by an aberrant TAM response promoting fibrosis. Despite limitations associated with small sample size, we have identified putative genes and pathways that may be involved in carcinogenesis. These data will inform future studies to elucidate the mechanisms driving AFX that will ultimately lead to prognostic biomarkers or tumor-directed therapy.

## Supporting information

S1 Fig PCA of AFX tumors. A PCA of AFX tumors (red), paired keratinocytes (blue), and 4 dermal derived fibroblasts (green).
(TIFF)

S2 Fig GO term enrichment analysis. The top 25 enriched pathways identified by GSEA from genes that are upregulated in AFX against keratinocytes and fibroblasts. The x-axis displays the number of genes in a particular GO term, whereas the label represents adjusted p-value.
(TIF)

S1 Table Somatic mutations identified in AFX samples (n = 8). (XLSX)

S2 Table Gained or deleted genes from the copy number variation analysis. (XLSX)

S3 Table Differentially expressed genes in common between AFX compared to both keratinocytes and fibroblasts. (XLSX)
