# Supplementary figures and images for: Genomic analysis of atypical fibroxanthoma
Source: PLoS One. 2017 Nov 15;12(11):e0188272. doi: 10.1371/journal.pone.0188272 (PMC5687749; doi:10.1371/journal.pone.0188272)

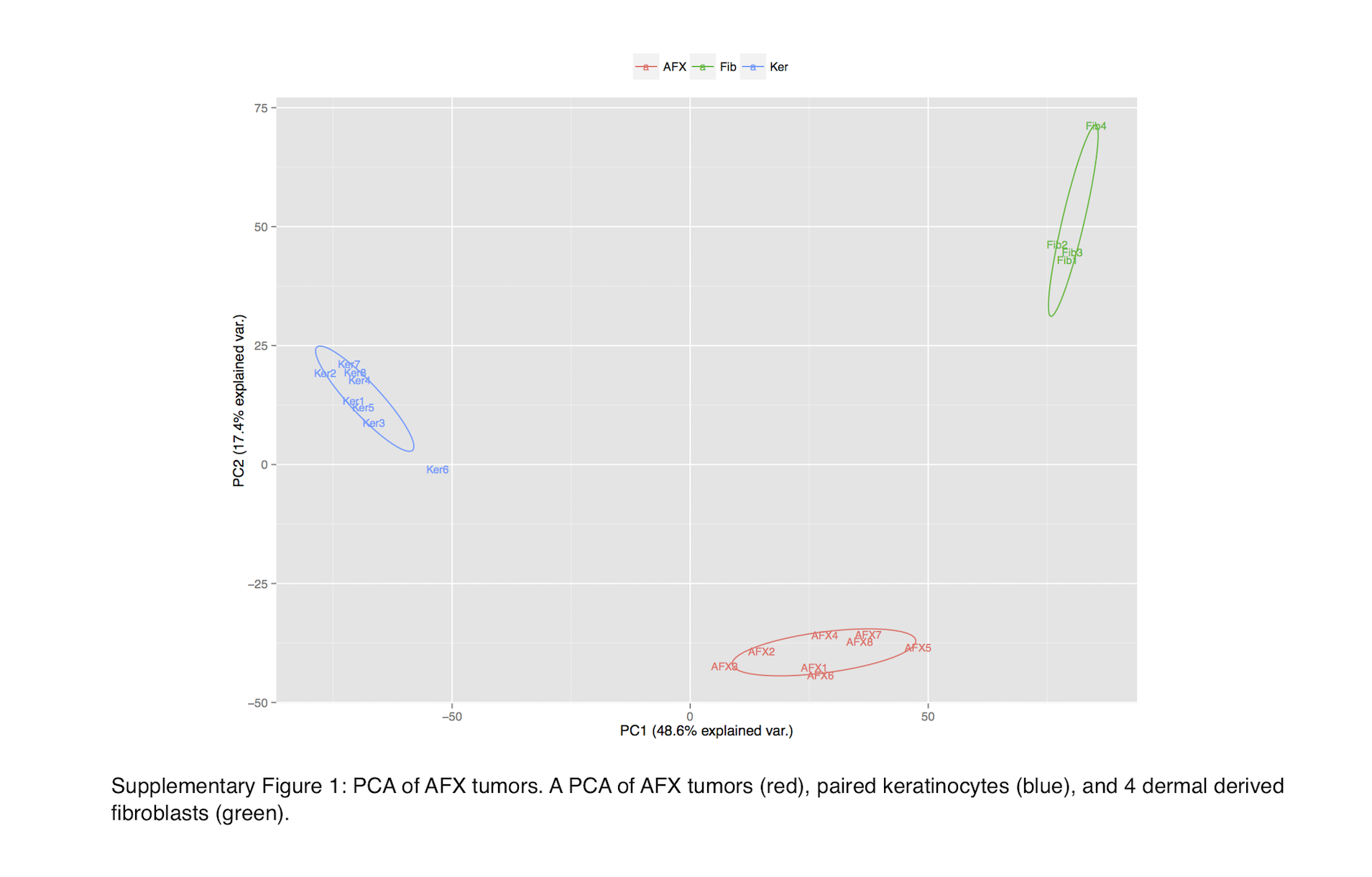

Supplement: S1 Fig — A PCA of AFX tumors (red), paired keratinocytes (blue), and 4 dermal derived fibroblasts (green). (TIFF) [file pone.0188272.s001.tiff]

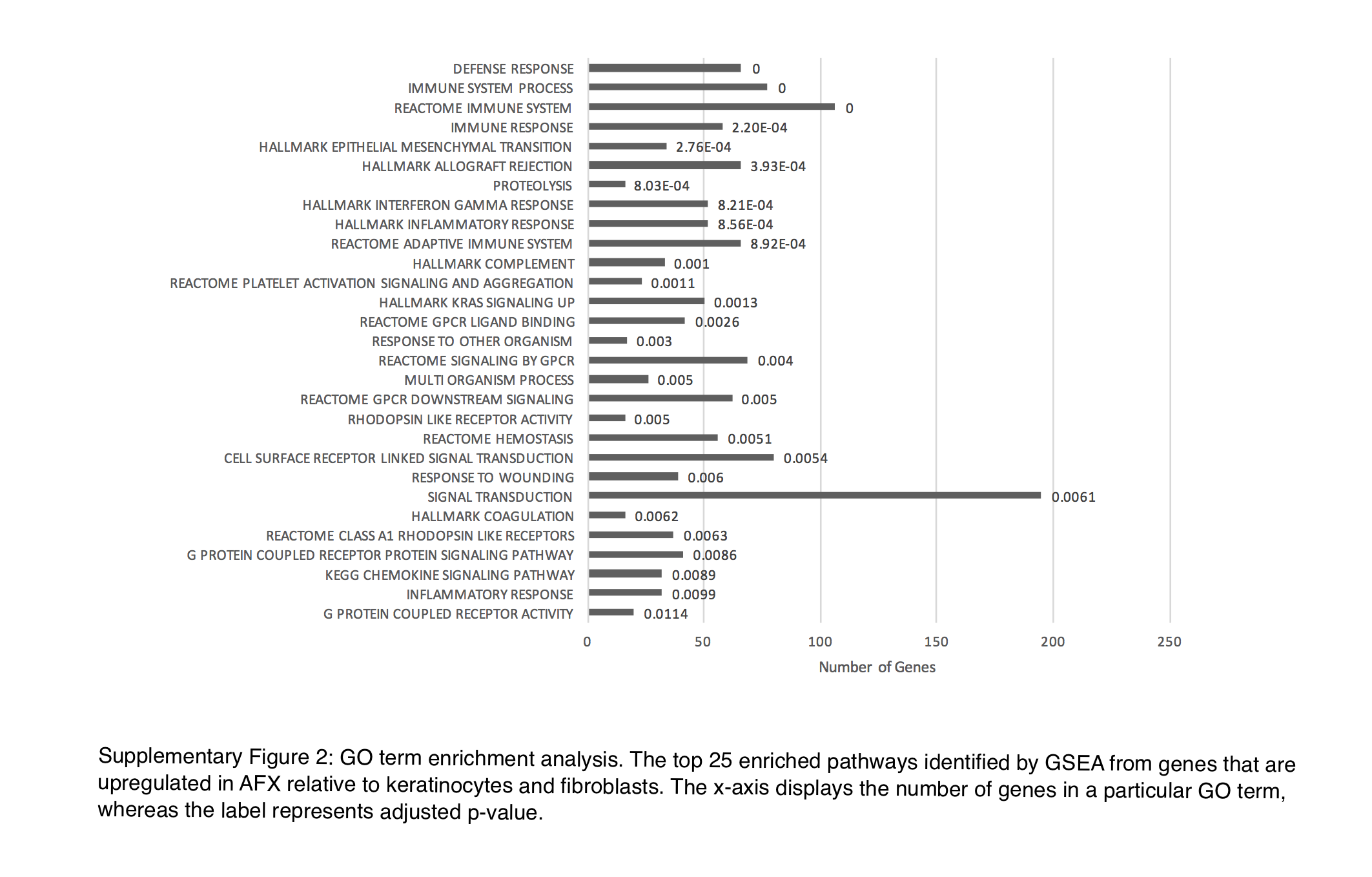

Supplement: S2 Fig — The top 25 enriched pathways identified by GSEA from genes that are upregulated in AFX against keratinocytes and fibroblasts. The x-axis displays the number of genes in a particular GO term, whereas the label represents adjusted p-value. (TIF) [file pone.0188272.s002.tif]
